# Sputum Detection of Predisposing Genetic Mutations in Women with Pulmonary Nontuberculous Mycobacterial Disease

**DOI:** 10.1038/s41598-018-29471-x

**Published:** 2018-07-27

**Authors:** Julie V. Philley, Kate L. Hertweck, Anbarasu Kannan, Barbara A. Brown-Elliott, Richard J. Wallace, Anna Kurdowska, Harrison Ndetan, Karan P. Singh, Edmund J. Miller, David E. Griffith, Santanu Dasgupta

**Affiliations:** 1 Department of Medicine, The University of Texas Health Science Center at Tyler, Tyler, Texas USA; 2 Department of Biology, The University of Texas at Tyler, Tyler, Texas USA; 3 Department of Cellular and Molecular Biology, The University of Texas Health Science Center at Tyler, Tyler, Texas USA; 4 Department of The Mycobacteria/Nocardia Research Laboratory Department of Microbiology, The University of Texas Health Science Center at Tyler, Tyler, Texas USA; 5 Department of Epidemiology and Biostatistics, The University of Texas Health Science Center at Tyler, Tyler, Texas USA; 6 Department of The Center for Heart and Lung Research, The Feinstein Institute for Medical Research, Manhasset, New York USA

## Abstract

Nontuberculous mycobacterial lung disease (NTM), including *Mycobacterium avium* complex (MAC), is a growing health problem in North America and worldwide. Little is known about the molecular alterations occurring in the tissue microenvironment during NTM pathogenesis. Utilizing next generation sequencing, we sequenced sputum and matched lymphocyte DNA in 15 MAC patients for a panel of 19 genes known to harbor cancer susceptibility associated mutations. Thirteen of 15 NTM subjects had a diagnosis of breast cancer (BCa) before or after NTM infection. Thirty three percent (4/12) of these NTM-BCa cases exhibited at least 3 somatic mutations in sputa compared to matched lymphocytes. Twenty four somatic mutations were detected with at least one mutation in *ATM*, *ERBB2*, *BARD1*, *BRCA1*, *BRCA2*, *AR*, *TP53*, *PALB2*, *CASP8*, *BRIP1*, *NBN* and *TGFB1* genes. All four NTM-BCa patients harboring somatic mutations also exhibited 15 germ line *BRCA1 and BRCA2* mutations. The two NTM subjects without BCa exhibited twenty somatic mutations spanning *BRCA1*, *BRCA1*, *BARD1*, *BRIP1*, *CHEK2*, *ERBB2*, *TP53*, *ATM*, *PALB2*, *TGFB1* and 3 germ line mutations in *BRCA1 and BRCA2* genes. A single copy loss of *STK1*1 and *AR* gene was noted in NTM-BCa subjects. Periodic screening of sputa may aid to develop risk assessment biomarkers for neoplastic diseases in NTM patients.

## Introduction

The incidence and prevalence of nontuberculous mycobacterial lung disease (NTM) is increasing in USA and worldwide^1–9^. In the USA, a prevalence of 20 NTM cases out of 100,000 in 1997 and 47 cases out of 100,000 in 2007 was observed with an 8.2% annual increase^8^. NTM lung disease in the U.S.A. is primarily caused by *Mycobacterium avium* complex (MAC) and poses considerable challenges in diagnosis and treatment^1–4^. NTM may occur in patients with or without a predisposing condition^1–9^. Postmenopausal women and individuals with various conditions such as bronchiectasis and cystic fibrosis are at risk of developing NTM disease^1–10^. Notably, the frequency of NTM patients with nodular bronchiectasis has increased over the past few years^8^. However, the underlying cellular and physiologic abnormalities causing NTM lung disease are not well understood^9^. In our recent study^8^, we observed that some women with stable NTM disease developed breast cancer (BCa) later in their lifetime, suggesting that NTM infection could be a potential risk factor for chronic inflammation and cellular transformation similar to *Helicobacter* pylori associated gastrointestinal transformation^11^. We rationalize that assessment of molecular abnormalities at infection sites and the surrounding environment would be important for continuous monitoring of the NTM infected patients. This will aid to develop suitable biomarkers for disease surveillance, treatment guidance and risk assessment.

Next generation sequencing is a powerful tool for detecting molecular abnormalities in tissues and body fluids and guiding biomarker and therapeutic development in various diseases including cancer. In the present study, on a next generation sequencing platform, we sequenced sputum and matched lymphocyte DNA in 15 women with NTM lung disease (MAC) for a panel 19 cancer predisposing genes^12–34^. Thirteen out of 15 NTM subjects had a diagnosis of BCa before or after NTM lung infection. Thirty three percent (4/12) of these NTM-BCa cases has been detected with 24 somatic mutations with at least one mutation in *ATM*, *ERBB2*, *BARD1*, *BRCA1*, *BRCA2*, *AR*, *TP53*, *PALB2*, *CASP8*, *BRIP1*, *NBN* and *TGFB1* genes. Numerous (N = 15) germ line *BRCA1 and BRCA2* mutations were also detected in these four subjects. The two NTM subjects with no history of BCa exhibited 20 somatic mutations spanning *BRCA1*, *BRCA1*, *BARD1*, *BRIP1*, *CHEK2*, *ERBB2*, *TP53*, *ATM*, *PALB2*, *and TGFB1 and* 3 germ line mutations in *BRCA1 and BRCA2* genes. In addition to genomic mutations, copy number loss in STK11, AR gene was evident in one NTM subject with BCa.

## Results

### Clinicopathological characteristics of the NTM affected women

Based on the available clinical history, thirteen out of the fifteen NTM patients we sequenced had also been diagnosed with BCa (Table 1). Notably, four out of these thirteen subjects were detected with NTM disease first and then BCa within a period of 4–6 years (Table 1). All the NTM subjects in our study cohort were positive for Bronchiectasis. Hormonal contraceptives were used by 67% (10/15) of the women with both NTM and BCa. In addition, 64% (9/14, no information available for 1 case) of these patients had undergone postmenopausal hormone therapy as well.

**Table 1:** Demographic information of the NTM-BCa and NTM subjects with the mutation spectrum and CNV.

NTM-BCa/NTM cases^a^	Age at NTM diagnosis	Age at BCa Diagnosis	BCa Stage	^b^Smoking/Drinking	Weight (lb)	Height (inches)	^c^BMI	^d^Somatic Mutation	^e^Germline Mutation	^f^CNV
1	75	38	UK	UK	120	63	21.3	N	N	Y
2	60	65	0	N/N	132	65	22.0	N	N	N
3	66	45	I	N/N	143	65	23.8	Y	Y	N
4	57	61	I	N/N	180	69	25.0	N	N	Y
5	71	75	0	N/N	120	64	20.6	N	N	N
6	73	68	II	N/Y	150	63	26.6	N	N	N
7	54	52	II	N/N	132	66	21.3	N	N	N
8	63	58	I	FS/N	120	67	18.8	N	N	Y
9	61	45	0	FS/Y	145	66	23.4	N	N	Y
10	55	51	III	N/N	98	61	18.5	N	N	Y
11	68	68	0	FS/N	156	54	37.6	Y	Y	Y
12	55	61	IB	N/Y	157	67	24.6	Y	Y	N
13	66	56	II	N/N	91	65	*15*.*1*	Y	Y	N
NTM01	49	NA	NA	N/Y	109	65	*18*.*1*	Y	Y	N
NTM03	58	NA	NA	Y/Y	114	66	*18*.*4*	Y	Y	N

^a^Rows 1–13: Women with both NTM and breast cancer; Rows 14–15 (NTM01/NTM03): Women with NTM disease only.

^b^Tobacco and alcohol usage history. UK: Unknown; FS: Former smoker; Y: Yes; N: No. ^c^BMI: Body mass index determined following NHLBI criteria: Underweight =  <18.5; Normal weight = 18.5–24.9; Overweight = 25–29.9; Obesity = BMI of 30 or greater. ^d^Mutation in sputum DNA compared to matched normal lymphocyte DNA; ^e^Mutation in both sputum and matched normal lymphocytes. ^f^Copy number variation in sputa compared to the matched normal lymphocytes.

### Pattern of the somatic genomic variants in the sputum of NTM subjects with breast cancer

**Figure 1:**
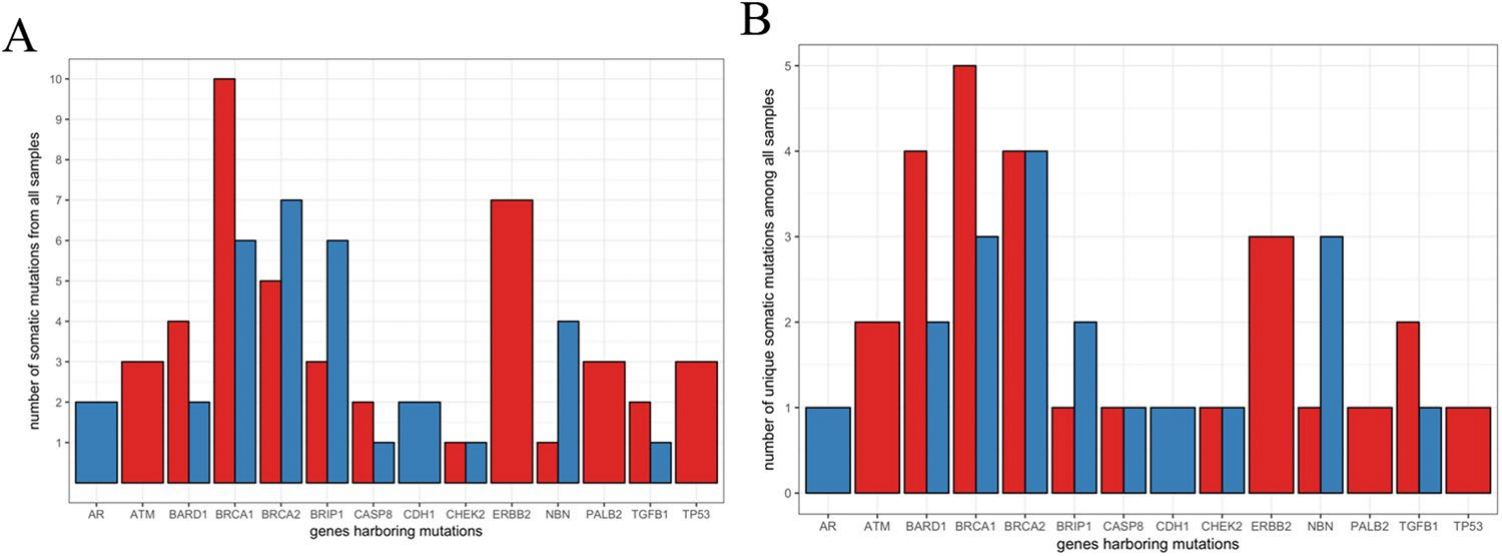
Nature of synonymous and nonsynonymous somatic mutations in women with pulmonary NTM disease. (**A**) Total somatic mutations tallied across all samples. Red bar represents missense mutations, blue bar represents synonymous mutations. (**B**) Unique somatic mutations in genes of interest from all samples (same data as (**B**), but mutations shared across samples are only counted once). Red bar represents missense mutations, blue bar represents synonymous mutations.

**Figure 2:**
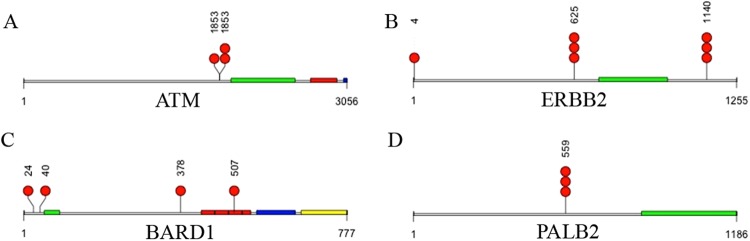
Nature of nonsynonymous (missense) somatic mutations in cancer associated genes *ATM* (**A**), *ERBB2* (**B**), *BRD1 (C) and PALB2* (**D**) in the NTM infected women. A red dot represents a mutation present in a single individual. The amino acid position affected by mutation was indicated above the red dot representing mutation. Multiple red dots indicate mutation present in more than one individual. Colored blocks indicate domains as described below for each gene. (**A**) *ATM* (Serine-protein kinase); green: FAT, red: Phosphatidylinositol 3- and 4-kinase, blue: FATC. Note: position 1853 possessed two different mutations. (**B**) BARD1 (BRCA associated RING domain 1); green: RING finger domain, red: ANK repeats, blue: BRCT1, yellow: BRCT2. (**C**) ERBB2 (Avian erythroblastic leukemia viral oncogene homolog 2); green: protein tyrosine kinase. (**D**) *PALB2* (Partner and localizer of BRCA2); green: WD40 repeat domain. The total length of the amino acids for each gene was also indicated below the domains.

**Figure 3:**
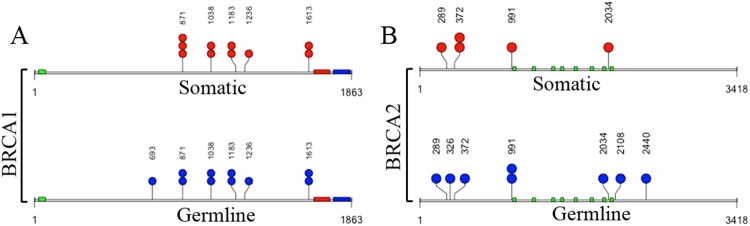
Nonsynonymous (missense) mutations in cancer associated genes in the sputum of NTM subjects. Red dots represent somatic mutations, blue dots represent germ line mutations in specific genes present in a single individual. The amino acid positions affected by mutations were indicated above the red or blue dots representing sequence variants. Multiple dots indicate mutation present in more than one individual. Colored blocks indicate domains as described below for each gene. (**A**) *BRCA1* (Breast cancer 1); green: RING finger domain, red: BRCT1 domain, blue: BRCT2 domain. (**B**) *BRCA2* (Breast cancer 2); green: *BRCA2* repeats. The total length of the amino acids for each gene was also indicated below the domains.

In this study, we have undertaken next generation sequencing (NGS) analysis of a 19-gene signature panel associated with cancer susceptibility and predisposition^12–34^ (Table 2) in women with NTM lung disease with (13) or without (two) a diagnosis of BCa. Matched lymphocytes and sputum DNA samples from thirteen NTM subjects with a history of BCa (NTM-BCa) and two subjects with NTM disease without BCa (NTM) were sequenced utilizing this high-throughput sequencing platform. Stringent data analysis and validation criteria were employed to determine both somatic and germ line mutations^35^. One subject did not pass quality control (NTM-BCa02) and was excluded from further analysis. Overall, we have detected numerous non-synonymous (Fig. 1A) and synonymous somatic mutations in these subjects (#PRJNA431897). Many unique somatic mutations were also identified in these samples (Fig. 1B). Thirty three percent (4/12) of the NTM subjects with a previous history of breast cancer (NTM-BCa) exhibited at least 3 somatic mutations in the sputum when compared to the matched lymphocytes (Table 2, Fig. 1). A total of 24 somatic mutations were detected in the sputum samples of these subjects with at least one mutation in *ATM*, *ERBB2*, *BARD1*, *BRCA1*, *BRCA2*, *TP53*, *PALB2*, *CASP8*, *BRIP1*, *NBN* and *TGFB1* gene. All the mutations were missense in nature (Table 3, Figs 1 and 2). We detected a novel *ERBB2* sequence variant (C-A, Ala > Glu; Table 3) in one NTM-BCa subject (NTM-BCa11), not reported previously. The genes most frequently harbored somatic mutations include *ERBB2* (N = 5), *BARD1* (N = 3), and *BRCA2* (N = 3) (Fig. 2). Notably, somatic mutations in *BRCA1* and *BRCA2* were detected in 75% (3/4) NTM-BCa subjects exhibiting mutations in the sputum (Table 3, Fig. 3).

**Table 2:** The panel of 19 cancer predisposing genes sequenced in the sputa of NTM affected women.

Gene	GenBank Reference	OMIM reference^a^	Description	CDDS (bp)^b^	Number of exons^c^
AR	NM_000044.3	313700	Androgen receptor	10661	8
ATM	NM_000051.3	607585	Serine-protein kinase ataxia telangiectasia mutated	13147	68
BARD1	NM_000465.3	601593	BRCA associated RING domain 1	5523	11
BRCA1	NM_007298.3	113705	Breast cancer 1, early onset	3699	22
BRCA2	NM_000059.3	600185	Breast cancer 2, early onset	11386	27
BRIP1	NM_032043.2	605882	BRCA1-interacting protein	8166	20
CASP8	NM_001080124.1	601763	Apoptosis-related cysteine protease 8	2750	9
CDH1	NM_004360.3	192090	Cadherin 1	4815	16
CHEK2	NM_001005735.1	604373	Serine/threonine checkpoint kinase 2	1991	16
DIRAS3	NM_004675.2	605193	GTP-binding Ras-like protein 3	1642	2
ERBB2	NM_001005862.1	164870	Avian erythroblastic leukemia viral concogene homolog 2	4816	30
NBN	NM_002485.4	602667	Nibrin	4639	16
PALB2	NM_024675.3	601355	Partner and localizer of BRCA2	4069	13
PTEN	NM_000314.4	601728	Phosphatase and tensin	5572	9
RAD50	NM_005732.3	604040	DNA repair protein AD50 homolog	6597	25
RAD51	NM_001164269.1	179617	DNA repair protein RAD51A homolog	2147	10
STK11	NM_000455.4	602216	Serine/threonine protein kinase 11	3286	10
TGFB1	NM_000660.4	190180	Transforming growth factor B1	2217	7
P53	NM_000546.5	191170	Tumor protein p53	2591	11

^a^Online Mendelian Inheritance in Man; ^b^CDDS: coding region of the genes in base pairs; ^c^Number of coding exons in each gene.

**Table 3:** Somatic mutations of the predisposing gene panel in NTM affected women with a diagnosis of breast cancer.

Sample^a^	Chromosome^b^	Gene^c^	Position^d^	Nature of Mutation	Reference^e^	LYM^f^	Sputum^g^	Amino acid Change^h^
NTM-BCa03	Chr11	*ATM*	108175463	Missense	A	A	T	Asp > Val
	Chr2	*BARD1*	215632255	Missense	C	C	T	Val > Met
	Chr2	*BARD1*	215674224	Missense	G	G	A	Pro > Ser
	Chr13	*BRCA2*	32906729	Missense	A	A	C	Asn > His
	Chr17	*BRIP1*	59763347	Missense	A	A	G	Ser > Pro
	Chr2	*CASP8*	202122995	Missense	A	A	G	Lys > Arg
	Chr17	*ERBB2*	37884037	Missense	C	C	G	Pro > Ala
	Chr17	*TP53*	7579472	Missense	G	G	C	Pro > Arg
NTM-BCa11	Chr2	*BARD1*	215674175	Missense	G	G	A	Ala > Val
	Chr17	*ERBB2*	37856502	Missense	C	C	A	Ala > Glu*
	Chr16	*PALB2*	23646191	Missense	T	T	C	Gln > Arg
NTM-BCa12	Chr17	*BRCA1*	41244936	Missense	G	G	A	Pro > Leu
	Chr13	*BRCA2*	32906729	Missense	A	A	C	Asn > His
	Chr13	*BRCA2*	32914592	Missense	C	C	T	Arg > Cys
	Chr17	*BRIP1*	59763347	Missense	A	A	G	Ser > Pro
	Chr17	*ERBB2*	37879588	Missense	A	A	G	Ile > Val
	Chr17	*ERBB2*	37884037	Missense	C	C	G	Pro > Ala
	Chr16	*PALB2*	23646191	Missense	T	T	C	Gln > Arg
NTM-BCa13	Chr11	*ATM*	108175463	Missense	A	A	T	Asp > Val
	Chr2	*CASP8*	202122995	Missense	A	A	G	Lys > Arg
	Chr17	*ERBB2*	37879588	Missense	A	A	G	Ile > Val
	Chr8	*NBN*	90990479	Missense	C	C	G	Glu > Gln
	Chr19	*TGFB1*	41858921	Missense	G	G	A	Pro > Leu
	Chr17	*TP53*	7579472	Missense	G	G	C	Pro > Arg

^a^NTM-BCa: Women with both NTM and a diagnosis of breast cancer; ^b^Chromosomal location of each of the gene exhibiting mutation; ^c^Name of the gene affected; ^d^Chromosome positions with reference to GRCh37.p13. ^e^Reference sequence of the human genome; ^f^Sequence detected in the lymphocytes DNA (LYM); ^**g**^Sequence detected in the sputum DNA; ^h^Corresponding change in the amino acid. An asterisk (*) indicates for a novel variant not reported previously.

### Pattern of somatic genomic variants in the sputum of women with NTM disease

Similar to the NTM patients with breast cancer history, both the NTM subjects (2/2, 100%) in the absence of breast cancer (Table 1) exhibited a number of somatic mutation in the sputum (Table 4, Figs 1 and 2). A total of 20 somatic mutations were detected in the sputum of these subjects with 11 mutations in one patient (NTM01) and 9 mutations in the other (NTM03) (Table 4). The majority of the somatic mutation were spanning *BRCA1* (N = 9) and *BRCA2* (N = 2) *and ERBB2* (N = 2) genes along with a single mutation in *BARD1*, *BRIP1*, *CHEK2*, *TP53*, *ATM*, *PALB2* and *TGFB1* molecules (Table 4, Figs 2 and 3). Two *ERBB2* gene mutations (chromosome position 37884037 and 37879588) that were detected in the NTM-BCa subjects (Table 3) were also present in the NTM subjects (Table 4). Similar was the case for mutations in *TP53* (chromosome position 7579472) and *PALB2* (chromosome position 23646191) for the NTM-BCa and NTM subjects (Tables 3 and 4).

**Table 4:** Somatic mutations of the predisposing genes in women with NTM infection in the absence of breast cancer.

Sample^a^	Chromosome^b^	Gene^c^	Position^d^	Nature of mutation	Reference^e^	LYM^f^	Sputum^g^	Amino acid Change^h^
NTM01	chr2	*BARD1*	215645464	missense	C	C	G	Arg > Ser
	chr17	*BRCA1*	41223094	missense	T	T	C	Ser > Gly
	chr17	*BRCA1*	41244000	missense	T	T	C	Lys > Arg
	chr17	*BRCA1*	41244435	missense	T	T	C	Glu > Gly
	chr17	*BRCA1*	41244936	missense	G	G	A	Pro > Leu
	chr13	*BRCA2*	32906480	missense	A	A	C	Asn > His
	chr13	*BRCA2*	32911463	missense	A	A	G	Asn > Asp
	chr17	*BRIP1*	59763347	missense	A	A	G	Ser > Pro
	chr22	*CHEK2*	29121019	missense	G	G	A	Arg > Cys
	chr17	*ERBB2*	37884037	missense	C	C	G	Pro > Ala
	chr17	*TP53*	7579472	missense	G	G	C	Pro > Arg
NTM03	chr11	*ATM*	108175462	missense	G	G	A	Asp > Asn
	chr17	*BRCA1*	41223094	missense	T	T	C	Ser > Gly
	chr17	*BRCA1*	41243840	missense	A	A	C	Asn > Lys
	chr17	*BRCA1*	41244000	missense	T	T	C	Lys > Arg
	chr17	*BRCA1*	41244435	missense	T	T	C	Glu > Gly
	chr17	*BRCA1*	41244936	missense	G	G	A	Pro > Leu
	chr17	*ERBB2*	37879588	missense	A	A	G	Ile > Val
	chr16	*PALB2*	23646191	missense	T	T	C	Gln > Arg
	chr19	*TGFB1*	41858876	missense	C	C	G	Arg > Pro

^a^NTM: Women with NTM disease only; ^b^Chromosomal location of each of the gene exhibiting mutation; ^c^Name of the gene affected; ^d^Chromosome positions with reference to GRCh37.p13. ^e^Reference sequence of the human genome; ^f^Sequence detected in the lymphocytes DNA (LYM); ^g^Sequence detected in the sputum DNA; ^h^Corresponding change in the amino acid.

### The spectrum of *BRCA1* and *BRCA2* germ line variants in the sputum of women with NTM-BCa

Germ line mutation in *BRCA1* and *BRAC2* genes are known risk factors for BCa development in women harboring mutation in these genes^25,28,32^. Other than somatic mutation, all the above described NTM-BCa patients (4/12, 33%) have exhibited a number of germline mutations in *BRCA1* and *BRCA2* genes (Table 5, Fig. 3). A total of 15 germ line *BRCA1* (N = 8) and *BRCA2* (N = 7) gene mutations were detected in these subjects (Table 4, Fig. 3). All the mutations were missense in nature. Thus, both somatic as well as germ line mutations in *BRCA1* and *BRCA2* genes were evident in the NTM infected women with BCa (Tables 3 and 5; Fig. 3). Notably, subject NTM-BCa11, a past smoker (14 years) who harbored a novel ERBB2 variant (Table 3) and germ line *BRCA2* mutation (Table 5), had a family history of BCa, and was diagnosed with BCa and NTM at the same age (Table 1).

**Table 5:** Distribution of germline mutations in BRCA1 and BRCA2 in women both NTM-BCa and NTM disease.

Sample^a^	Chromosome^b^	Gene^c^	Position^d^	Nature of mutation	Reference^e^	LYM^f^	Sputum^g^	Amino acid Change^h^
NTM-BCa03	Chr17	*BRCA1*	41223094	missense	T	C	C	Ser > Gly
	Chr17	*BRCA1*	41244000	missense	T	C	C	Lys > Arg
	Chr17	*BRCA1*	41244435	missense	T	C	C	Glu > Gly
	Chr17	*BRCA1*	41244936	missense	G	A	A	Pro > Leu
	Chr17	*BRCA1*	41245471	missense	C	T	T	Asp > Asn
	Chr19	*BRCA2*	32906593	missense	C	A	A	Ser > Arg
NTM-BCa11	Chr13	*BRCA2*	32906480	missense	A	C	C	Asn > His
	Chr13	*BRCA2*	32911463	missense	A	G	G	Asn > Asp
NTM-BCa12	Chr17	*BRCA1*	41223094	missense	T	C	C	Ser > Gly
	Chr17	*BRCA1*	41244000	missense	T	C	C	Lys > Arg
	Chr17	*BRCA1*	41244435	missense	T	C	C	Glu > Gly
	Chr13	*BRCA2*	32914815	missense	G	A	A	Arg > His
	Chr13	*BRCA2*	32929309	missense	A	G	G	His > Arg
NTM-BCa13	Chr13	*BRCA2*	32906729	missense	A	C	C	Asn > His
	Chr13	*BRCA2*	32914592	missense	C	T	T	Arg > Cys
NTM01	Chr17	*BRCA1*	41243840	missense	A	C	C	Asn > Lys
	Chr13	*BRCA2*	32906729	missense	A	C	C	Asn > His
NTM03	Chr17	*BRCA1*	41245471	missense	C	T	T	Asp > Asn

^a^NTM-BCa: Women with NTM disease and breast cancer, NTM: Women with NTM disease; ^b^Chromosomal location of each of the gene exhibiting mutation; ^c^Name of the gene affected; ^d^Chromosome positions with reference to GRCh37.p13. ^e^Reference sequence of the human genome; ^f^Sequence detected in the lymphocytes DNA (LYM); ^g^Sequence detected in the sputum DNA; ^h^Corresponding change in the amino acid.

### Distribution of *BRCA1* and *BRCA2* germ line variants in the sputum of NTM affected women

We also detected 3 germline mutation spanning *BRCA1* and *BRCA2* genes in both the NTM subjects (2/2, 100%) (Table 5, Fig. 3). Among the 3 germ line mutation, 2 were similar to that observed in the NTM patients with breast cancer history (chromosome position: 41245471, B*RCA1*; 32906729, *BRCA2*). However, the germ line mutation in *BRCA1* (position: 41243840) was only detected in the NTM subject in the absence of BCa (Table 4).

### Copy number variation (CNV) in different genomic and chromosomal regions in the NTM affected women

**Figure 4:**
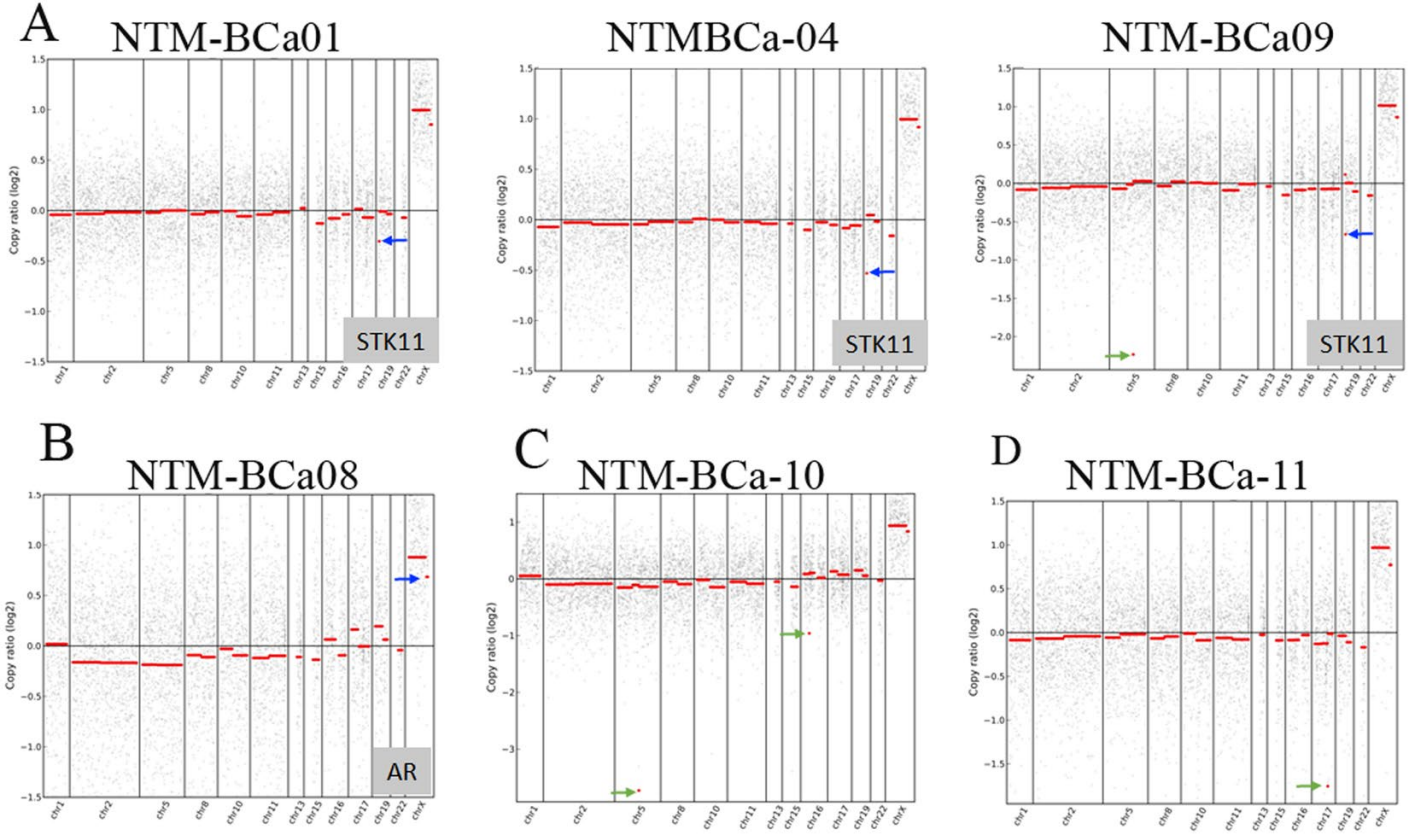
Copy number variation in NTM affected patients. (**A**) Single copy loss of *STK11* (named in gray box) as indicated by blue arrows in 3 subjects with NTM and breast cancer (NTM-BCa). (**B**) Copy number loss of androgen receptor (*AR*) gene (named in gray box), indicated by blue arrow in one subject with NTM and breast cancer (NTM-BCa). (**C**) Copy number loss in chromosomal region 5 and 16 (green arrows) in a woman with NTM and breast cancer (NTM-BCa). (**D**) Copy number loss in chromosome 17 (green arrow) in another woman with NTM and breast cancer (NTM-BCa).

The CNV analysis did not detect any breakpoints within genes for any samples (data not shown). No single sample contained more than one CNV in a target gene, although gains and losses in copy number periodically occurred throughout the rest of the genome. Lowering the CNV log2 call threshold to 0.2 (the default option in CNVkit) resulted in additional gains and losses of some chromosomal segments (data not shown), but only the high-confidence calls from a more stringent log2 threshold of 0.3 were presented. A single copy loss of a lung and breast cancer risk associated gene *STK11* (a.k.a. LKB1) was noted in 25% (3/12) of the NTM subjects with breast cancer history (Fig. 4A). A single copy loss of the Androgen Receptor gene (*AR*) was also noted in one NTM subject with BCa history (Fig. 4B). A single copy loss in chromosome 5 and 16 was noted in BCa-NTM10 (Fig. 4C) and copy number loss in chromosome 17 was noted in subject BCa-NTM11 (Fig. 4D) who had a history of breast cancer and smoking as described above. These CNVs observed in chromosome 5, 16 and 17 were not associated with the 19 gene panels we have analyzed (Fig. 4C).

## Discussion

Pulmonary NTM disease (especially disease due to MAC) is a rising health concern in USA and throughout the world^8^. Many of these NTM patients develop therapeutic resistance posing significant challenges to disease management^1,9^. Infection with MAC particularly in the treatment resistant patients may lead to molecular changes associated with inflammation and tumorigenesis in surrounding epithelial tissues niche. In a recent study, infection of normal human lung airway epithelial cells with MAC triggered enhanced expression of CCL20, IL-32 and CXCL8 proteins^36^. These proteins are known to promote BCa growth, invasion and progression to metastasis^37–43^. These molecules were also demonstrated to promote lung cancer^40,43–47^. A recent study also uncovered functional involvement of intratumoral pathogenic bacteria in facilitating chemotherapeutic resistance in colon cancer patients^48^. Thus, NTM infected patients may remain at risk of developing neoplastic disease in their life time. Investigation of the molecular genetic alterations in the surrounding tissue microenvironment of the infected sites is important and could aid in developing disease monitoring and risk assessment strategies.

Next generation sequencing platform has revolutionized characterization of molecular genetic abnormalities resulting from infection or genotoxic damages in various affected cell types^49–52^. Free circulating DNA released from the infected or malignant cells often serves as monitoring/surveillance biomarkers and can also offer better therapeutic guidance^53–55^. Increased sputum production is one of the major symptoms due to NTM lung infection and sputum could be a valuable resource to identify free DNA not only associated with NTM pathogenesis but also inflammatory changes resulted from infection. Free circulating DNA associated with altered methylation, inflammation and cancer has been detected in sputum of COPD and lung cancer patients^56–60^. We identified cancer associated predisposing genetic mutations in sputum of women with NTM lung infection with or without a diagnosis of BCa. This novel finding confirms the presence of mutated DNA in sputum samples of NTM infected patients. Numerous genes exhibiting somatic mutations in these subjects such as *EBBB2*, *PALB2*, *TP53*, *ATM*, *STK11* and *TGFB1* are involved in various malignancies including BCa^16,18,23,61^. The majority of the women in our study cohort had been diagnosed with BCa before or after the diagnosis of NTM disease. Patient 11 (NTM-BCa11), a former smoker with a high BMI and family history of breast cancer was simultaneously diagnosed with NTM and early stage BCa (stage 0) at age 68. This patient was detected with a novel somatic *ERBB2* mutation and germ line *BRCA2* mutation accompanied by copy number loss in chr.17. Similarly, patient 12 (NTM-BCa-12) with a history of alcohol consumption who was diagnosed with NTM (MAC) at age 55 and stage-IB breast cancer at age 61 exhibited numerous somatic and germ line *BRCA1* and *BRCA2* mutations in the sputum. The two NTM subjects with low BMI and exhibiting somatic/germ line *BRCA1* and *BRCA2* mutations had presented with abnormal mammograms during their routine examination. Except for subject NTM01, they also had a history of alcohol consumption and tobacco smoking. Collectively, these findings suggest an association between NTM (MAC) lung disease and BCa development in these women and warrants breast examinations and routine screening of sputum for predisposing mutation detection.

*STK11* (a.k.a. LKB1) is a critical regulator of mammary tumorigenesis^62–64^. Functional inactivation or loss of STK11 was shown to promote breast cancer initiation and progression to metastasis^62–64^. A functional coordination between *STK11* with *ERBB2* in mediating these effects in mammary tumorigenesis was also demonstrated^62–64^. The STK11 is also one of the most frequently inactivated genes in non-small cell lung cancer^65^. Loss of *STK11* copy in multiple NTM-BCa subjects also suggests association with malignant transformation in these women. Notably, one of these women with *STK11* alteration (NTM-BCa04) was diagnosed with NTM disease at age 57 and stage-I breast cancer at 61. These findings further suggest a functional correlation between NTM and malignant disease development in these women. Androgen receptor (*AR*) expression predicts better prognosis and survival of breast cancer patients^34^ and reduced *AR* expression promotes initiation of *ERBB2* induced mammary tumorigenesis^66^. Thus, the loss of *AR* copy number observed in one NTM-BCa subject could also be associated with neoplastic transformation in this subject.

Germ line pathogenic variants in *BRCA1* and *BRCA2* are predisposing genetic factors associated with enhanced risk of BCa in the lifetime of an individual as demonstrated in numerous studies^12,25,26,28,31–33^. In this study, 40% of NTM affected women irrespective of their BCa diagnosis status exhibited germ line *BRCA1* and *BRCA2* mutations. Thus, NTM patients with long term infections and predisposing cancer associated genetic mutations may be at risk of developing malignant diseases in their life-time. Comparing the number of somatic mutations between the NTM-BCa (4/13) and the NTM (2/2) groups, there was no statistically significant relationship between breast cancer status and somatic mutation (p = 0.06) among NTM patients. Similarly, no statistically significant relationship was established for NTM-BCa with germ line (p = 0.06) mutation and copy number variations (p = 0.214).

To our knowledge, this is the first study, which revealed cancer associated gene mutations (both somatic and germ line) in sputum of NTM (MAC) infected subjects with or without a diagnosis of breast cancer. These findings suggest that chronic infection with NTM may trigger inflammation and cellular transformation surrounding the infection sites (immune and epithelial cells). Therefore, these subjects may potentially be at risk of acquiring transformational changes due to chronic NTM infection. Earlier, we have detected oncogenic ECM1 protein in the circulating exosomes of these subjects^8^. These findings collectively suggest for an increased risk of the NTM affected subjects towards oncogenic transformation. *Helicobacter pylori* infection is a relevant example facilitating gastrointestinal tumorigenesis^11^. This study suggests a novel avenue for study and may warrants monitoring of these subjects not only for NTM progression but also cellular transformation. In the clinical setting, molecular assessment of sputa by high throughput sequencing of NTM affected subjects may identify novel genetic alterations. A consensus panel of such molecular alterations could serve as biomarker for monitoring the risk of developing neoplastic disease in these patients as do *BRCA1*, *BRCA2 and ER/PR/HER2* biomarkers for BCa^25–27,52^.

## Methods

### Human samples and ethical statement

Matched normal lymphocytes and sputa with relevant clinical information such as age, grade, stage, diagnosis etc. were collected from 13 NTM-BCa and 2 NTM subjects (de-identified, Table 1). The Institutional Review Board of The University of Texas Health Science Center at Tyler approved this study (#974). All subjects had sputum cultures which were culture positive for MAC infection by acid fast bacilli (AFB) sputum analysis^8^. Informed consent was obtained from all the patients. All methods were performed in accordance with the relevant guidelines and regulations.

### Sputum collection and quality control

Routine expectorated sputa were collected and cultured as necessary for detection of AFB^67–69^. Samples were processed using standard decontamination procedures, fluorochrome microscopy and cultured on solid and liquid media as recommended by the Clinical and Laboratory Standards Insititute (CLSI) guidelines for mycobacteria detection and culture^67,69^. MAC isolates were identified using AccuProbe (Hologic Gen-Probe Inc)^68^. For decontamination, the N-acetyl-L-cysteine-sodium hydroxide method alone or in combination with oxalic acid was used^70^. All methods were performed in accordance with relevant guidelines and regulations.

### DNA extraction and quantification

The lymphocytes were isolated from whole blood as described^71^. Genomic DNA was extracted from lymphocytes and sputa by digesting samples with 1% sodium dodecyl sulfate/proteinase K mixture overnight at 55 °C. The DNA was then extracted by phenol-chloroform, and ethanol precipitation and suspended in Tris-EDTA buffer and concentration was measured using the Nanodrop System. For sequencing analysis, 1 µg of DNA was used.

### Next generation sequencing of the predisposing human gene panel

Utilizing next generation sequencing (NGS) platform^35^, we sequenced sputum and matched lymphocyte DNA of 15 NTM subjects for a panel 19 genes known to harbor mutations associated with cancer susceptibility and neoplastic transformation (Table 2)^12–34^. A total of 313 exons (coding regions) covering 63619 base pair regions of these 19 genes were mapped. We utilized a custom oligonucleotide-based capture with sequencing of regions within these 19 genes on Illumina HiSeq platform.

### Data analysis and validation

The analysis pipeline utilizes genome analysis tool kit (GATK) standards, includes quality assessment with FASTQC^72^ followed by mapping reads to human reference genome GRCh37.p13 (hg19). SNVs and indels with depth of coverage >10 were called using Burrows-Wheeler Aligner (BWA)^73^ and Sequence Alignment/Map (SAM) tools^74^, with annotation from the Human Genetic Mutation Database using SnpEff^75^. When multiple annotations for gene location were available, the most severe was reported (e.g., missense variant scored instead of non-coding exon variant). Following Winter *et al*.^32^, variants were classified as somatic if they were present only in the sputa; and germ line if they were present in both the lymphocyte and sputa compared to the reference sequence. Variants present only in the lymphocytes were excluded from subsequent analysis. Sequence data have been submitted to the NCBI Sequence Read Archive and can be found under accession (BioProject #PRJNA431897). Scripts used for filtering and visualizing results can be found at https://github.com/k8hertweck/breastCancerNTM.

### Copy number variation analysis

CNVs were assessed using CNVkit^76^. This software uses both target (e.g., from cancer associated genes) and off-target reads to call copy number across the genome, and is most accurate in detecting CNVs larger than 1 mega base pair (Mbp) spanning multiple exons (or captured regions). Log2 values for segment calls were summarized for visualization purposes. Given the uncertainty in assessing levels of copy number heterogeneity associated with these samples, the log2 threshold of 0.3 (as recommended by the CNV kit manual) was applied to call gains or losses in copy number of target genes.

### Statistical analysis

We employed Binomial test for proportion to compare mutation outcome among various groups.
